# Utilizing the arts to improve health, resilience, and well-being (HeRe We Arts^®^): a randomized controlled trial in community-dwelling individuals with chronic medical conditions

**DOI:** 10.3389/fpubh.2024.1242798

**Published:** 2024-02-07

**Authors:** Lisa Gallagher, Tamara Shella, Debbie Bates, Isaac Briskin, Maria Jukic, Francois Bethoux

**Affiliations:** ^1^Arts and Medicine, Cleveland Clinic, Cleveland, OH, United States; ^2^Department of Physical Medicine and Rehabilitation, Neurological Institute, Cleveland Clinic, Cleveland, OH, United States; ^3^Quantitative Health Sciences, Cleveland Clinic, Cleveland, OH, United States

**Keywords:** arts, arts in health, arts integration, health, resilience, well-being, music therapy, art therapy

## Abstract

**Background:**

Healthcare workers are concerned with promoting behavior changes that enhance patients’ health, wellness, coping skills, and well-being and lead to improved public health. The purpose of this randomized controlled trial was to determine if participation in an 8-week arts-based program leads to improved mood, health, resilience, and well-being in individuals with chronic health conditions as compared to a wait list control group.

**Methods:**

Self-report questionnaires for well-being, mental health, physical health, overall health, social health, mood, coping, and resilience were administered at baseline, Week 8 (end of program), and Week 16 (8-week follow-up).

**Results:**

Statistically significant improvements were noted in all outcome measures for the treatment group, as well as in most areas compared to the control group. Many of the positive results at Week 8 were either maintained or further improved at Week 16.

**Discussion:**

These results suggest that arts-based programming can have a positive effect on the mood, health, resilience, and well-being of individuals with chronic health conditions. Therefore, arts-based programming should be utilized more frequently in the management of chronic conditions in community-dwelling individuals. These benefits should be further assessed in larger clinical trials.

## Introduction

Healthcare professionals increasingly focus on the promotion of behavior changes to improve their patients’ health, well-being, and coping skills. Specific areas of focus include chronic health conditions, mental health, physical health, aging, obesity, and unhealthy lifestyles (1–3). These issues have led to increased attention to racial migration, an aging population, rate of growth in population, economic and social effects of the population as it ages, movement to rural areas from urban areas, frequent hospital readmissions, inadequate care, and increased behavioral health needs (4, 5). Due to the wide variety of concerns, and individuals facing these concerns, it is important to find multiple ways of addressing them as it is unlikely that one specific method would be effective for everyone.

The World Health Organization in 1948 defined health as “a state of complete physical, mental, and social well-being and not merely the absence of disease or infirmity” (6, 7). Therefore, health encompasses multiple domains (emotional, physical, occupational, intellectual, spiritual, and social). Well-being has been defined as an individual’s perception of physical health, improved symptoms, and psychological functioning (8). Resilience has been defined as individuals’ abilities and/or characteristics that help them to bounce back, recover from challenges, cope with adversity, and manage stress (9–12). If the ability to cope is negatively impacted, the imbalance in homeostasis that is caused could also lead to physical and mental health issues (10, 11).

Individuals suffering from chronic health conditions are impacted in physical, mental, and social areas of their lives as they attempt to deal with various stressors, learn new ways to cope, and improve their resilience (13). A growing number of treatment programs are offered to help promote self-care, adherence to physical activity, manage anxiety, stress, and depression, and optimize quality of life and optimism.

Programs have been created to help with recovery from illness and improving individuals’ resilience while facing multiple challenges (9, 10, 14–17). Some of these programs utilize the arts. While many were single interventions that focused on just one art modality (e.g., visual arts, crafting, music, drumming, writing, movement, or theater) throughout the course of the program or study, others combined multiple artistic interventions or mixed art with more traditional health promotion interventions.

We designed the HeRe We Arts^®^ program to include multiple arts interventions over the course of several weeks so that the participants could be presented with a variety of experiences. The use of multiple interventions or strategies is also known as bundling (18), and the bundling approach has been used in health-related research (19, 20). Therefore, we believed that multiple interventions should be used that focused on individual needs, as well as those of the community, family and society as individuals have different strengths, abilities, preferences, and learning styles (21–23). Our goal was to intervene in the context of a population health initiative, targeting community-dwelling individuals with a wide spectrum of personal and medical situations and challenges, with the overall goal of reducing health inequities, working with a variety of settings for intervention delivery. In this context, integrating complementary arts-related components into the program was our preferred strategy (23).

This study was designed to address the broad problem of revising current health programs while addressing the need to promote wellness, population health, public health, and prevention (1–3, 5). The specific problem to be addressed was revising current health programs to help improve the health, resilience, well-being, and mood of adults coping with chronic health conditions (2, 17–30). At the time of this study, there was limited literature regarding the use of arts-based interventions utilizing various styles of learning to help improve resilience, well-being, health, and mood in outpatient settings (21–23, 25, 31–40).

The purpose of this randomized controlled trial (RCT) was to determine if participation in an 8-week arts-based program, delivered in a group format within an outpatient community setting, would lead to improved health, resilience, mood, and well-being in adults with chronic health conditions as compared to similar individuals in a wait-list control group. An arts-based approach was chosen as it was believed to be non-threatening, inexpensive, and something familiar, safe, and comfortable that would engage participants of all ages, genders, backgrounds, ethnicities, and abilities. The primary theory that informed this study was arts integration theory (AIT). AIT is a means of teaching topics, meeting objectives, and engaging in creative processes through various arts experiences (41). These interventions are provided by trained individuals in order to connect the skills from the arts to other subjects (42). This study was designed to expose individuals to multiple arts experiences in the hope that they would find at least one that resonated with them and that they would engage in in the future. It was also hoped that support and social relationship would assist with the improvement of the coping skills, behavior changes, and health outcomes (43–47).

Specifically, this study aimed at assessing within-and between-group differences at end of intervention (Week 8) and 8-week follow-up (Week 16) on mood, well-being, resilience, perceived health status, and self-reported physical activity. We hypothesized that participants in the treatment group would demonstrate within-group improvement, and greater improvement compared to the control group, for all outcome measures at Week 8, and that these improvements would be maintained at Week 16.

## Materials and methods

### Setting

The study was conducted at two local urban community health and education centers (Langston Hughes Community Health & Education Center and Stephanie Tubbs Jones Health Center), one suburban family health center (Lakewood Family Health Center), and one local urban hospital (Akron General Hospital) within a single healthcare system. These locations were chosen because their administrators were creatively working on addressing the health needs of members of their local communities, many of which were considered to be underserved neighborhoods where individuals were dealing with chronic health conditions that were causing many doctor visits and hospital readmissions. IRB-approved fliers about the program were posted at and shared with individuals attending these locations. Health care providers, employees, and volunteers at these locations assisted with the recruitment of participants by sharing the flier, discussing the study, and/or referring individuals to call the number on the flier if interested in participating. Large community rooms, education rooms, conference rooms, gyms, and/or auditoriums were where each of the weekly sessions were held.

### Participants

Participants were recruited from areas surrounding the local community health and education centers and/or hospital. The inclusion criteria were: at least 18 years old; diagnosed with at least one chronic health condition (as reported by the participant) for which health promotion and maintenance were recommended; able to participate safely in all program sessions; proficient in English; and cognitively able to consent to participate. Exclusion criteria were severe visual or auditory impairment; and severe and/or uncontrolled comorbidity precluding safe participation in a physical activity program.

### Study design

Individuals who expressed interest in participating in the study were screened by study personnel either in person or by phone using a pre-determined script that was based on the inclusion/exclusion criteria. If the individual was deemed eligible to participate, study personnel met with the individual, explained the study, reviewed the informed consent form, and obtained written informed consent. Randomization then occurred based on an online random number generator (48). This tool works differently every time it is used as it is set to the time of the computer’s clock (48). Each participant was assigned to either the treatment group or a wait-list control group based on the blocks assigned by the random number generator. Spouses, family, or friends needing to accompany a disabled participant were also invited to participate if they met the inclusion/exclusion criteria. The treatment group attended the first set of sessions that were offered, whereas the control group would not attend the sessions until they were offered the following time, which occurred at least 12–16 weeks after the first round of sessions. Similar information regarding involvement in arts experiences were collected for both groups.

### Ethics statement

Written informed consent was obtained for all participants in this study, and the privacy rights of human subjects were observed. This study was approved by the hospital’s Institutional Review Board (IRB) study #17–1732. The work described here was carried out in accordance with The Code of Ethics of the World Medical Association (Declaration of Helsinki) for experiments involving humans. Data were collected and managed using REDCap electronic data capture tools hosted at [Vanderbilt University] (49, 50). REDCap (Research Electronic Data Capture) is a secure, web-based software platform designed to support data capture for research studies. Data were de-identified to protect confidentiality and anonymity. This study was considered minimal risk by our IRB.

### Intervention

Each week of the 8-week arts-based program included an educational component and an experiential component. All of these components were designed to incorporate different learning styles and abilities of the participants, many of which related to Gardner’s Theory of Multiple Intelligences (21). These are listed below for each week. Personnel involved in the various sessions included the hospital’s art and music therapists and art curators, as well as music therapists from Beck Center for the Arts, a local community arts partner. All of them were trained to the study protocol and program curriculum. Although many of the sessions were led by music or art therapists, the program was designed such that it could be led by creative arts therapists, artists, musicians, actors, writers, and/or healthcare providers with an arts background. Each session was approximately 2 h in length, allowing time for socialization and refreshments, didactic learning, and active participation. A small stipend (USD 10) was provided to study participants at every session to help cover their expenses and time, including testing sessions for the control group. This helped some participants with paying for their transportation. An additional payment of USD 20 was sent after the completion of the questionnaires at Week 16. Participants who missed sessions were kept in the study, unless they requested to withdraw. Study personnel also called the participants at times to touch base and to remind them of the next week’s session.

The following is an outline of the 8-week program; however, at times weeks 2 through 7 were conducted in a different order based upon space requirements and presenter availability.

#### Week 1

Introduction to the Arts and Health was an introduction to the 8-week series of courses, and an introduction to the connection between the arts and health, as well as to the concepts of well-being and resilience. An interactive art experience was utilized, with participants creating a talisman key chain to represent themselves and their desires for the program. Intelligences addressed: linguistic–verbal, logical-mathematical, visual–spatial, body-kinesthetic, and intrapersonal (21).

#### Week 2

Music, Well-Being, and Resilience was a session that provided information on how music could elicit positive physical and emotional responses. The social aspects of music were discussed as were the benefits of listening to music. Interactive music interventions were utilized, including lyric discussion, singing, instrument playing, and music-assisted relaxation techniques. Intelligences addressed: musical, body-kinesthetic, interpersonal, and intrapersonal (21).

#### Week 3

Movement and Physical Activity was a session that included information on the importance of physical activity in improving mood, health, resilience, and well-being; as well as the emotional release that could occur. Discussion was held on how pairing the arts (particularly music) with physical activity could increase interest in, and length of, the activity. Interactive movement and drumming exercises based on the Drums Alive^®^ program were utilized. Drums Alive^®^ is a program that utilizes rhythm, music, and physical fitness to create physical, emotional, social, and mental well-being (51). Intelligences addressed: body-kinesthetic, interpersonal, and visual–spatial (21).

#### Week 4

Art and Well-Being was a session that included discussion of how artmaking could be utilized to promote healthy habits, resilience, well-being, self-care, and self-expression. Participants were encouraged to create a collage representing themselves on the front of a journal that would later be used during the Writing and Communication/Self-Expression week. Pictures, words, phrases, etc. from magazines, scrapbook supplies, decorative objects, and mod podge were utilized to decorate the covers of the journals. Participants were encouraged to share about their journal covers with the other participants if they were willing to do so. Intelligences addressed: visual–spatial, body-kinesthetic, and intrapersonal (21).

#### Week 5

Writing and Communication/Self-Expression was a session in which the participants utilized the journals they created in a previous session. Information was provided on the importance of communication and self-expression to one’s health, resilience, and well-being. A variety of poetry, journaling, storytelling, and song-writing techniques were taught and practiced. Participants were encouraged to share from their journals as they felt comfortable. Intelligences addressed: linguistic–verbal, and intrapersonal (21).

#### Week 6

Theater and Socialization was a session in which information was provided on the importance of interaction, support, and understanding of others to one’s mood, health, resilience, and well-being. Interactive theater games were utilized and discussions regarding attending, and volunteering at, theater productions were held. Discussions were also included regarding stepping out of one’s comfort zone and on the importance of socialization. Intelligences addressed: linguistic–verbal, body-kinesthetic, and interpersonal (21).

#### Week 7

Art Appreciation and a Healthy Brain was a session that included information on the effects of visual arts on cognition, emotion, learning, and memory. Examples of uses of music to improve brain functioning were also shared. Various visual art forms, especially surrounding public art, were shared and discussed; and participants engaged in a small group art-making experience. During this experience they were encouraged to utilize provided supplies that included pictures of sculptures, markers, colored pencils, scissors, etc. to create their own sculpture garden. Many created a theme for their garden and shared the art they created with the other groups. Intelligences addressed: naturalistic, body-kinesthetic, logical-mathematical, interpersonal, and visual–spatial (21).

#### Week 8

Summary/Integration of the Arts into Daily Lives included an integration of the knowledge and skills learned in all the other sessions. These were summarized and discussed, and participants were encouraged to share how they had been using the arts outside of the sessions. They were also reminded to continue to utilize the arts in their daily lives to promote mood, health, resilience, and well-being after the completion of the program. The session ended with a group drumming experience. Intelligences addressed: linguistic–verbal, logical-mathematical, musical, and interpersonal (21).

### Data collection and outcome measures

Data collected included participants’ demographic information (age, gender, race/ethnicity, location of group, medical diagnoses, comorbidities), goals, and responses to measurement tools. The dependent variables that were addressed included mood, health, resilience, well-being, physical activity, and behavior change. Data were collected on all of these variables except behavior change through the use of the following standardized questionnaires: Godin-Shephard Leisure Time Physical Activity Questionnaire (GSLTPAQ), Short Depression-Happiness Scale (SDHS), Short Warwick-Edinburgh Mental Well-Being Scale (SWEMWBS), PROMIS Scale v1.2 – Global Health, and Brief Resilient Coping Scale (BRCS). Behavior change was measured on a weekly basis through following up with participants to ask if they completed the items they listed on their Weekly Take-Away Forms. Data were collected on the independent variable of arts-based programming through the post-session surveys that were provided at the end of each session, as well as the Pre/Post-Test HeRe We Arts^®^ Survey.

At Week 1 all pre-test and baseline assessments were conducted, the Weekly Take-Away Form was completed, and the participants filled out the Weekly Post-Session Survey. Prior to the start of the sessions for Weeks 2–7 each participant was individually asked if they completed the Take-Away from the previous week, and at the end of the sessions the Weekly Post-Session Survey and Weekly Take-Away Form were completed. During Week 8 all post-tests and final 8-week surveys were conducted and prior to the start of the session each participant was individually asked they completed the Take-Away from the previous week. Individuals in the control group completed the same assessments as the experimental group at Weeks 1, 8, and 16.

The instruments listed below were used in a feasibility study that was conducted prior to the original randomized controlled trial. They were found to be short, easy to use, and easy to understand. Some could be completed in five (5) minutes, but none of them took longer than 20 min to complete. Permission was obtained for the various instruments that required permissions; however, many were in the public domain or were available for use for research.

The Godin-Shephard leisure-time physical activity questionnaire (GSLTPAQ) was utilized to measure physical activity. This questionnaire asks participants how many times on average, over a 7-day period, they engage in strenuous, moderate, or mild exercise for more than 15 min, and the average frequency of activity that leads to increased heart rate (52). Rationale for use included the short length of the scale, the ease of understanding it, and its ability to characterize the level of physical activity of the participants.

The Short Depression-Happiness Scale (SDHS) was used to assess mood and overall well-being. It is based on the Depression-Happiness Scale (DHS), but it is designed to take a shorter amount of time, as well as to provide a means of assessing change while keeping the completion of self-report measures to a minimum. It contains 6 items, 3 negative and 3 positive. The negative items include: I feel dissatisfied with my life, I felt cheerless, and I felt that life was meaningless (53). The positive items include: I felt happy, I felt pleased with the way I am, and I felt that life was enjoyable (53). Individuals completing this questionnaire are asked to think about how they felt in the past 7 days and to rate the frequency of item on a 4-point scale. Rationale for selection included the scale being short, it was easy to understand, and it allowed the researchers to assess participants’ mood (depression and happiness) which is essential to their health and well-being.

The Short Warwick-Edinburgh Mental Well-Being Scale (SWEMWBS) was utilized to assess participants’ well-being. It asks participants to answer 7 questions by choosing the answer that best describes their experience over the last 2 weeks. Ratings include none of the time, rarely, some of the time, often, or all the time (54, 55). This test was designed to measure the feeling and functioning aspects of positive mental well-being. Rationale for selection included the scale being short, easy to understand, and its ability to assess well-being.

The PROMIS Scale v1.2—Global Health was utilized to assess health. It is a self-report measure to identify symptoms, feelings, behaviors, and functions in the areas of physical, mental, and social health (56). The rationale for use included the scale being fairly short and easy to understand, as well as its ability to assess global health and well-being.

The Brief Resilience Coping Scale (BRCS) was utilized to measure coping or resilience. It is a 4-item measure designed to identify participants’ abilities to cope with stress; and it may be helpful for recognizing those participants who may need to learn techniques to help improve their coping skills and resilience (57). Based on their scores on this scale, participants are identified as low resilient copers, medium (average) resilient copers, or high resilient copers (57, 58). Rationale for use included the scale being short, easy to understand, and its ability to assess resilience.

In addition to these validated and reliable measures, the researchers in the original study created a pre-test/post-test HeRe We Arts^®^ Survey to test knowledge on arts and well-being, as well as satisfaction at endpoints. In addition, to improve knowledge and promote behavioral changes, at the end of each week’s session participants were asked to complete the following sentence on a Take-Away Form: “I plan to use ___________ at least once this week in order to improve my health and well-being” on a typed sheet of paper that they took home. Research personnel took a picture of this statement on their encrypted Cleveland Clinic iPhone. These photos were then downloaded onto a secure drive and kept in the participants’ research folders. The researcher guiding the study followed up with them the next week to learn if they had completed their take-away from the previous week. Finally, at the end of each session, the participants completed a Weekly Post-Session Survey to obtain information on learning and satisfaction.

### Quantitative and qualitative analyses

Data analysis included the total number of participants enrolled, the number (%) of participants attending the sessions, and the number (%) of participants withdrawing from or removed from the study. Descriptive statistics were generated on the responses to the satisfaction questionnaires. Assessment included whether participants were successful in implementing the strategies in the short term (during the program), and in continuing to implement the strategies 8 weeks (or 2 months) after program completion, based on a 3-level rating (1 – Fully; 2 – Partially; 3 – Not at all). Paired *t-*tests were used to test for change over time on outcome measures within the experimental group, two-sample *t*-tests were used to test for difference results between the experimental group and the control group, with *p* < 0.05 considered statistically significant. No adjustment was made for multiple comparisons, owing to the fact that this was a pilot RCT. Qualitative coding analysis were used to obtain information from any open-ended questions on the surveys. Mean changes were calculated from baseline with 95% confidence intervals in measured scales. Imputations were used and a value was assigned to data that was missing. Missing data was minimized by reviewing it for completion when it was submitted by the participants; however, some data was still missing or not completed by some of the participants.

Prior to the start of the study a G* Power analysis, power 0.80 and effect size 0.25, was conducted in order to determine the number of participants needed to demonstrate statistical significance. At that time, it was estimated that 128 individuals would be needed, with 64 assigned to each group.

## Results

### Participants

**Figure 1:**
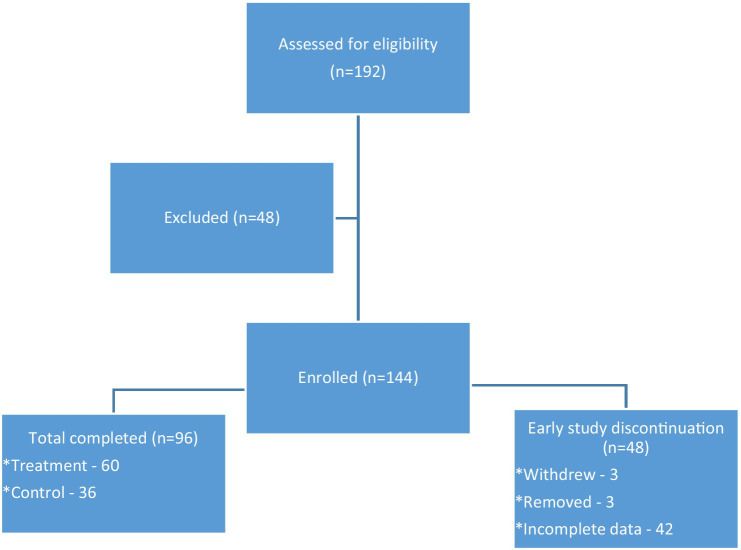
The enrollment flow diagram demonstrating eligibility, exclusion, enrollment, completion, and early study discontinuation.

See Figure 1 for the CONSORT Flow Diagram. A total of 192 participants were assessed for eligibility, and 48 of those screened were removed due to exclusion criteria. Of the 144 remaining, 48 more were removed due to inability to continue participating, withdrawal from the study, lack of participation, or lack of completing follow-up questionnaires. In the end, the final numbers in the control group and treatment group were not evenly distributed.

Of the 96 who completed the study, 36 were in the control group and 60 were in the treatment group. The age range of participants was 18–91 years with the average age of the participants being 61.5 years. A total of 89.6% of participants were female, almost 60% were African American or Black, and over one third had high school degrees or less. See Table 1 for descriptive statistics and a summary of the cohort characteristics. Participants had at least one diagnosed chronic health condition, but some had more than one. Those listed most frequently were hypertension (59.4%), overweight or obese (54.2%), rheumatism or arthritis (46.9%), neck or back pain (39.6%), pre-diabetes or diabetes (26.0%), depression (22.9%), breathing or lung problems (21.9%), anxiety (20.8%), chronic pain (20.8%), and heart condition (11.5%).

**Table 1:** Descriptive statistics.

Variable	Level	[ALL]	N
Randomized group	Control group	36 (37.5%)	96
	Experimental Group	60 (62.5%)	
Age		62.0 [55.0; 69.0]	96
Gender	Female	86 (89.6%)	96
	Male	10 (10.4%)	
Race/Ethnicity	Black/African American	57 (59.4%)	96
	White/Caucasian	31 (32.3%)	
	Other	8 (8.33%)	
Highest Degree/Level of School Completed			
	High school or less	33 (34.4%)	96
	Associate or technical degree	23 (24.0%)	
	Bachelor’s degree	25 (26.0%)	
	Graduate degree	15 (15.6%)	
Employment Status	Employed	21 (21.9%)	96
	Self-Employed	4 (4.17%)	
	Homemaker	1 (1.04%)	
	Student	2 (2.08%)	
	Retired	48 (50%)	
	On Disability	13 (13.5%)	
	Unemployed	7 (7.29%)	
Baseline SDHS		14.0 [11.0; 16.0]	96
Baseline SWEMWBS		26.0 [23.8; 29.2]	96
Baseline BRCS		15.0 [13.0; 17.0]	96
Baseline Godin Activity Score		22.5 [15.0; 42.2]	96
Baseline PROMIS Mental T Score		44.6 [41.1; 50.8]	96
Baseline PROMIS Physical T Score		42.3 [37.4; 50.8]	96
(At baseline) In general, would you say your health is	Poor/Fair	29 (30.2%)	96
	Good	50 (52.1%)	
	Very Good/Excellent	17 (17.7%)	
(At baseline) In general, please rate how well you carry out your usual social activities and roles…			
	Poor/Fair	23 (24.0%)	96
	Good	36 (37.5%)	
	Very Good/Excellent	37 (38.5%)	
Arthritis/rheumatism	No	51 (53.1%)	96
	Yes	45 (46.9%)	
Back or neck pain	No	58 (60.4%)	96
	Yes	38 (39.6%)	
Heart condition	No	85 (88.5%)	96
	Yes	11 (11.5%)	
Diabetes	No	71 (74.0%)	96
	Yes	25 (26.0%)	
Chronic pain	No	76 (79.2%)	96
	Yes	20 (20.8%)	
Hypertension/high blood pressure	No	39 (40.6%)	96
	Yes	57 (59.4%)	
Lung/breathing problem	No	75 (78.1%)	96
	Yes	21 (21.9%)	
Anxiety	No	76 (79.2%)	96
	Yes	20 (20.8%)	
Cancer	No	91 (94.8%)	96
	Yes	5 (5.21%)	
Stroke	No	94 (97.9%)	96
	Yes	2 (2.08%)	
Depression	No	74 (77.1%)	96
	Yes	22 (22.9%)	
Obesity/overweight	No	44 (45.8%)	96
	Yes	52 (54.2%)	

### Quantitative data

There was statistically significant improvement in the treatment group from Weeks 1 to 8 for SDHS, SWEMWBS, BRCS, PROMIS Mental T Score, PROMIS Physical T Score, PROMIS Q1, and PROMIS Q9 and from Weeks 1 to 16 for SDHS, SWEMWBS, BRCS, PROMIS Mental T Score, PROMIS Physical T Score, PROMIS Q1, and PROMIS Q9 (Table 2). Table 2 also reports between-group differences in mean change scores. There was a statistically significant difference favoring the treatment group results from Weeks 1 to 8 for SDHS, SWEMWBS, PROMIS Mental T Score, PROMIS Physical T Score, PROMIS Q1, and PROMIS Q9. From Weeks 1 to 16 there were significant differences for SWEMWBS, PROMIS Mental T Score, PROMIS Physical T Score, and PROMIS Q9.

**Table 2:** Change in outcome measures.

Variable	Treatment group	Control group	Within-group	Between-group
	Mean difference (95% CI)	Mean difference (95% CI)	*p* values	*p* values
Baseline to Week 8				
SDHS	1.43 (0.74, 2.13)	0 (−0.94, 0.94)	***<0.001***	***0.016***
SWEMWBS	1.8 (0.87, 2.73)	0 (−1.15, 1.15)	***<0.001***	***0.016***
BRCS	1 (0.09, 1.91)	0.14 (−0.71, 0.99)	***0.031***	0.166
Godin Activity Score	5.42 (−2.11, 12.95)	0.86 (−6.81, 8.53)	0.155	0.395
PROMIS Mental T Score	4.08 (2.54, 5.61)	1.33 (−0.19, 2.85)	***<0.001***	***0.012***
PROMIS Physical T Score	3.12 (1.82, 4.41)	0.15 (−1.22, 1.51)	***<0.001***	***0.038***
PROMIS Q1	–	–	***<0.001***	***0.005***
PROMIS Q9	–	–	***<0.001***	***0.002***
Baseline to Week 16				
SDHS	0.97 (0.34, 1.6)	0.17 (−0.73, 1.07)	***0.003***	0.146
SWEMWBS	1.6 (0.76, 2.44)	−0.64 (−1.48, 0.2)	***<0.001***	***<0.001***
BRCS	1.27 (0.49, 2.04)	−2.06 (−8.89, 4.78)	***0.002***	0.133
Godin Activity Score	0.08 (−7.65, 7.81)	0.48 (−1.46, 2.42)	0.983	0.677
PROMIS Physical T Score	2.5 (1.41, 3.59)	0.5 (−1.06, 2.06)	***<0.001***	***0.038***
PROMIS Q1	–	–	***0.015***	0.066
PROMIS Q9	–	–	***<0.001***	***0.012***

The bold values represent *p* values that are statistically significant.

Table 3 demonstrates a summary of the results specific to each research question and hypothesis. Participants who engaged in the HeRe We Arts^®^ program also completed pre-and post-surveys regarding their use of various art forms and whether they believed that their health and/or well-being could improve by participating in arts-based programming. Overall, participants engaged in more art forms after the program, and 98.6% as compared to 76% indicated that they felt participating in arts-based programming could improve their health and/or well-being (Table 4). In addition to the questionnaires listed above, at the completion of each session participants completed post-session survey data (Table 5). This indicated the percentage of change in their stress, anxiety, and mood during each session; as well as how they rated the helpfulness of the session. The sessions rated the highest (very good and/or excellent) included Movement, Writing, and Theater. During the Introduction, Movement, Art Appreciation, Theater, and Summary sessions 100% of participants indicated that their stress, anxiety, and mood got better or stayed the same.

**Table 3:** Research questions, measures, hypotheses, and outcomes.

Research question	Items measured	Questionnaire	Hypotheses	Outcomes
Q1	Mood & well-being	SDHS	Treatment group will have improved mood and well-being when comparing Week 8 to participants’ own baseline Treatment group will have better mood and well-being than control group at Week 8 Treatment group will have improved mood and well-being when comparing Week 16 to participants’ own baseline Treatment group will have better mood and well-being than control group at Week 16	Supported Supported Supported Not supported
Q2	Well-being	SWEMWBS	Treatment group will have improved well-being when comparing Week 8 to participants’ own baseline Treatment group will have better well-being than control group at Week 8 Treatment group will have improved well-being when comparing Week 16 to participants’ own baseline Treatment group will have better well-being than control group at Week 16	a. Supported b. Supported c. Supported d. Supported
Q3	Resilience	BRCS	Treatment group will have improved resilience when comparing Week 8 to participants’ own baseline Treatment group will have better resilience than control group at Week 8 Treatment group will have improved resilience when comparing Week 16 to participants’ own baseline Treatment group will have better resilience than control group at Week 16	a. Supported b. Not supported c. Supported d. Not supported
Q4	Health & well-being	PROMIS Mental Health T Scores	Treatment group will have improved health and well-being when comparing Week 8 to participants’ own baseline Treatment group will have better health and well-being than control group at Week 8 Treatment group will have improved health and well-being when comparing Week 16 to participants’ own baseline Treatment group will have better health and well-being than control group at Week 16	a. Supported b. Supported c. Supported d. Supported
Q5	Health	PROMIS Physical Health T Scores	Treatment group will have improved health when comparing Week 8 to participants’ own baseline Treatment group will have better health than control group at Week 8 Treatment group will have improved health when comparing Week 16 to participants’ own baseline Treatment group will have better health than control group at Week 16	a. Supported b. Supported c. Supported d. Supported
Q6	Health & well-being	PROMIS Question 1 Overall Impression Scores	Treatment group will have improved health and well-being when comparing Week 8 to participants’ own baseline Treatment group will have better health and well-being than control group at Week 8 Treatment group will have improved health and well-being when comparing Week 16 to participants’ own baseline Treatment group will have better health and well-being than control group at Week 16	a. Supported b. Supported c. Supported d. Not supported
Q7	Health & well-being	PROMIS Question 9 Social Health Scores	Treatment group will have improved health and well-being when comparing Week 8 to participants’ own baseline Treatment group will have better health and well-being than control group at Week 8 Treatment group will have improved health and well-being when comparing Week 16 to participants’ own baseline Treatment group will have better health and well-being than control group at Week 16	a. Supported b. Supported c. Supported d. Supported
Q8	Health	GSLTPAQ	Treatment group will have improved health when comparing Week 8 to participants’ own baseline Treatment group will have better health than control group at Week 8 Treatment group will have improved health when comparing Week 16 to participants’ own baseline Treatment group will have better health than control group at Week 16	a. Not supported b. Not supported c. Not supported d. Not supported

**Table 4:** HeRe We Arts^®^ pre-post survey results (treatment group only—Week 1 to Week 8).

Question	Pre-survey	Post-survey	% Change
	*N* = 75	*N* = 74	
	[n (%)]	[n (%)]	
Do you think by participating in arts-based programming you can improve your health and/or well-being?			
Yes	57 (76)	73 (98.6)	
Do not Know	18 (24)	1 (1.4)	
	*N* = 85	*N* = 57	
Art forms currently used			
Art (viewing)	27 (36)	43 (58.1)	+59%
Art (creating)	26 (34.7)	38 (51.4)	+35%
Dance/movement (viewing)	26 (34.7)	29 (39.2)	+12%
Dance/movement (active participation)	22 (29.3)	32 (43.7)	+45%
Music (attending performances)	31 (41.3)	21 (28.4)	−32%
Music (listening to)	56 (74.7)	50 (67.6)	−11%
Music (playing instrument/singing)	19 (25.3)	24 (32.4)	+26%
Theater (attending performances)	28 (37.3)	28 (37.8)	−0%
Theater (performing)	4 (5.3)	6 (8.1)	+50%
Writing	26 (34.7)	54 (73)	+108%

**Table 5:** Post-session specific survey data.

Question	Intro [n (%)]	Music [n (%)]	Art [n (%)]	Writing [n (%)]	Movement [n (%)]	Art appreciation [n (%)]	Theater [n (%)]	Summary [n (%)]
Overall, how would you rate this session?	*N* = 69	*N* = 69	*N* = 68	*N* = 72	*N* = 68	*N* = 67	*N* = 70	*N* = 64
Excellent	44 (63.8)	56 (81.2)	51 (75.0)	54 (75.0)	58 (85.3)	49 (73.1)	59 (84.3)	53 (82.8)
Very good	19 (27.5)	10 (14.5)	13 (19.1)	16 (22.2)	9 (13.2)	13 (19.4)	9 (12.9)	9 (14.1)
Good	5 (7.2)	2 (3.0)	4 (6.0)	2 (3.0)	1 (1.0)	4 (6.0)	1 (1.0)	2 (3.0)
Fair	1 (1.0)	1 (1.0)	0	0	0	1 (1.0)	1 (1.0)	0
Poor	0	0	0	0	0	0	0	0
How helpful was the content presented?	*N* = 69	*N* = 69	*N* = 68	*N* = 72	*N* = 68	*N* = 67	*N* = 68	*N* = 64
Extremely helpful	37 (53.6)	47 (68.1)	48 (70.6)	53 (73.6)	56 (82.4)	47 (70.1)	55 (80.9)	50 (78.1)
Very helpful	28 (40.6)	20 (29)	19 (27.9)	16 (22.2)	10 (14.7)	14 (20.9)	11 (16.2)	14 (21.9)
Somewhat helpful	3 (4.0)	2 (3.0)	1 (1.0)	3 (4.0)	2 (3.0)	5 (7.0)	1 (1.0)	0
Not so helpful	1 (1.0)	0	0	0	0	1 (1.0)	1 (1.0)	0
Not at all helpful	0	0	0	0	0	0	0	0
Did you notice any change in your level of stress during the session?	*N* = 69	*N* = 69	*N* = 68	*N* = 72	*N* = 68	*N* = 67	*N* = 70	*N* = 63
Got better	48 (69.6)	58 (84.1)	59 (86.8)	55 (76.4)	62 (91.2)	53 (79.1)	64 (91.4)	54 (85.7)
Stayed the same	21 (30.4)	10 (14.5)	8 (11.8)	16 (22.2)	6 (9.0)	14 (21.0)	6 (9.0)	9 (14.3)
Got worse	0		1 (1.0)	1 (1.0)	0	0	0	0
N/A	0	0	0	0	0	0	0	0
Did you notice any change in your level of anxiety during the session?	*N* = 69	*N* = 68	*N* = 67	*N* = 72	*N* = 68	*N* = 67	N = 70	*N* = 63
Got better	40 (58.0)	53 (77.9)	51 (76.1)	53 (73.6)	57 (83.8)	50 (74.6)	58 (82.9)	50 (79.4)
Stayed the same	29 (42.0)	14 (20.1)	15 (22.4)	18 (25.0)	11 (16.2)	17 (25.4)	12 (17.1)	13 (20.6)
Got worse	0	1 (1.0)	1 (1.0)	1 (1.0)	0	0	0	0
N/A	0	0	0	0	0	0	0	0
Did you notice any change in your mood during the session?	*N* = 68	*N* = 69	*N* = 68	*N* = 72	*N* = 68	*N* = 66	*N* = 70	*N* = 63
Got better/increased	54 (79.4)	57 (82.6)	59 (86.8)	57 (79.2)	60 (88.2)	54 (81.8)	64 (91.4)	54 (85.7)
Stayed the same	14 (20.6)	10 (14.5)	9 (13.2)	14 (19.4)	8 (11.8)	12 (18.2)	6 (8.6)	9 (14.3)
Worse/decreased	0	2 (3.0)	0	1 (1.0)	0	0	0	0
N/A	0	0	0	0	0	0	0	0

### Qualitative data

Due to the large amount of quantitative data obtained during this study, the qualitative data obtained via questionnaires and/or semi-structured interviews with the participants will be addressed in a separate manuscript. However, we would like to share some of the comments made by participants:

- “It meant so much to me that you took the time to learn and use our names.”
- “I have the power to do things to help me relax; it’s up to me.”
- “I came out of my dark spot and now deal with the real journey of life.”
- “It got me out of the house, and I’ve made some new friends.”
- “This is the first thing I ever signed up for that I finished.”
- “I can be more than my pain.”

## Discussion

In this RCT comparing the HeRe We Arts^®^ program to wait-list controls, we observed statistically significant improvements in the treatment group at the end of the program on most outcome measures (except for physical activity on the GSLTPAQ), which was sustained 8 weeks later. We also found statistically significant between-group differences favoring the treatment group on all of the PROMIS scales, SDHS, and SWEMWBS at Week 8, and on PROMIS Mental Health, PROMIS Physical Health, PROMIS Q9, and SWEMWBS at Week 16.

The GSLTPAQ was the only score that did not significantly improve with treatment. This may be due in part to the fact that a longer intervention is necessary to promote change in physical activity. Only one session of the program directly addressed physical activity. In addition, participants may need one-on-one coaching to understand how to overcome individual barriers to physical activity. Therefore, arts-based programming may be coupled with more traditional physical activity programs to achieve optimal results.

It was hypothesized that the change in BRCS scores would be significantly greater for the treatment group than for the control group; however, this was not the case, although there was a statistically significant improvement at Week 8 and Week 16 within the treatment group. This finding may be related to insufficient sample size and needs to be further investigated. Additional content directly related to resilience may need to be added to the program curriculum.

Participant survey results indicated an increase in the belief that participating in arts-based programming could help improve well-being and/or health as responses to this question changed from 76% prior to the program to 98.6% after. There also appeared to be an increase in the percentage of change for the use of various art forms such as creating and viewing art (+35% and + 59%), singing or playing instruments (+26%), performing theater (+50%), participating in and viewing dance (+45% and + 12%), and writing (108%). Possibilities for the greatest increase in writing was that it may have previously been the least familiar of the various art forms or the fact that participants may have engaged in this more frequently because they provided with a journal that they decorated during the Art and Well-Being session and were urged to use outside of sessions.

Individuals who participated in the HeRe We Arts^®^ program seemed to have a high level of satisfaction with the program. Responses on the Post-Session Survey demonstrated that 98.6% were very or extremely likely to recommend the program, 97.3% rated the sessions as very good or excellent, and 98.6% rated the sessions as very or extremely helpful. Although it is possible that participants felt they needed to rate everything positively, they were encouraged on multiple occasions to be honest. It is believed that participants were honest in their responses as noted by the fact that some sessions were marked as only somewhat helpful or not so helpful or only as good or fair. Participants also marked if their anxiety, stress, and/or mood stayed the same or got worse. Therefore, if they responded honestly to some questions, it is believed that they responded honestly to all questions.

Recommendations for future research include the utilization of a larger sample size. This will allow for the possibility of generalization of results, as well as for more statistically significant results. It is further recommended that there be more of an attempt to equally randomize the size of the treatment and the control groups. As a follow-up to this, it may be valuable to engage the control group earlier by providing them with another type of program so that they stay involved and complete the study. It could also be helpful to include a measurement of social relationships as this appeared to be a valuable component for participants. This was noted in the quantitative and qualitative data. Finally, it is recommended that the 8-week HeRe We Arts^®^ program be compared to another type of 8-week group such as a health education program. This might not only assist with follow-through and increasing engagement, it might also help to control for the Hawthorne Effect and help to truly determine the usefulness of arts-based programming for improving health outcomes.

### Contributions to practice

Although it is possible to make recommendations for clinical practice based on the results of this study, due to the inability to meet the required number of participants it is not possible to generalize these results to other populations. As population health continues to be an area of concern, and there continues to be an aging population, programs like HeRe We Arts^®^ could be a beneficial means of improving the health, resilience, mood, and well-being of individuals. Improvement was noted in all of these areas for individuals dealing with chronic health conditions; and it is also possible that programs like this could be helpful for individuals coping with various mental and physical health problems. Programs utilizing the arts are fairly inexpensive, enjoyable, accessible, and effective. They can help provide education about health topics while engaging participants in something they can easily do on their own or with others to improve their health, resilience, well-being, coping, and socialization. This is especially important after the pandemic when isolation, loneliness, quality of life, health, well-being, cognition, and socialization became challenges facing many people, but especially older adults and those with chronic health conditions (59–61). The following recommendations for practice are suggested based on the results found in this study:

1. Consider utilizing multiple arts interventions within one program. This allows for different learning styles, preferences, abilities, and strengths of participants while addressing multiple behaviors at once (21–23, 26, 39, 62–65).
2. Utilize arts integration theory as the theoretical framework for clinical practice and programming. Individuals participated in experiential and didactic learning, and they were encouraged to utilize what they learned outside of sessions, share it with their families and friends, and integrate the use of the arts into their lives. To our knowledge, this is one of the first studies to utilize multiple arts forms and arts integration theory in population health to address well-being, health, resilience, and mood in individuals with chronic health conditions. Therefore, continuing to use this theory will help to expand the theoretical framework in this area.
3. Utilize arts integration theory and arts-based programs to assist with improving resilience and coping skills. Previous research on resilience has demonstrated that as teamwork developed and therapeutic relationships occurred, psychological health and stress improved (66). Therefore, programs such as HeRe We Arts® could be models for accomplishing this.
4. Consider using arts integration theory and arts-based programming to assist with improving the health of those living with chronic health conditions. The results of this study demonstrated improved anxiety, stress, mood, well-being, resilience, and physical and mental health outcomes; therefore, more programs like this should be considered. A unique aspect of HeRe We Arts^®^ was that it was offered in various communities, some of which were in underserved, lower-income neighborhoods where individuals often do not have access to mental health support, medical care, healthy foods, or programs to help improve their health and coping skills. Many of the HeRe We Arts^®^ participants thanked the researchers for providing them with new skills, learning their names, caring about them, and bringing the program to their community.
5. Utilize creativity and arts-based programs to assist with improving well-being of participants, while at the same time educating these individuals on the importance of well-being. This is also in line with previous research which hypothesized that mental well-being could be improved through participating in arts interventions (31, 33).
6. Consider utilizing arts-based programming to address mental health needs such as stress, depression, anxiety, emotional well-being, and mood. The results of this study indicated that mood, anxiety, and stress improved for the majority of participants during each session. This is consistent with previous research that hypothesized that arts experiences could help promote mental well-being and improve mental health (31, 33, 67).
7. Utilize community arts-based programming to increase socialization. Arts-based programming has been found to improve social identity, personal and social well-being, socialization, and physical and mental health while also creating social connectedness and the opportunity to create new friendships (1, 33, 38, 67–69).
8. Utilize arts-based programming as a means of changing behaviors. This study demonstrated that many of the participants changed their behaviors during the 8 weeks of the program, and that they also either maintained this change or continued to improve on the changes for up to 2 months after the completion of the program. These changes in behaviors could improve various health indicators, act as preventative measures, and possibility even keep patients out of the hospital.

### Limitations of the study

The major limitation with this study is that the sample size did not reach the level that was identified as needed to determine statistical significance. Some of this was due to excluding so many potential participants at the beginning of the study (42), as well as excluding another 48 for withdrawing from or not completing the study. Therefore, lack of follow-through was a definite limitation to the study. The limited number of dropouts in the treatment group, compared to the wait-list control group, suggests that the feasibility and tolerability of the program were good. Lack of an active control group could also be considered a limitation. However, we chose to use the wait list control design for this initial study, with the intention to conduct another study later utilizing a RCT design with an active control group. Another limitation was the lack of generalizability. This includes the inability to generalize to a broad population of individuals, to all individuals dealing with chronic health conditions, or to persons in non-urban settings. Another limitation was the randomization process as some of it occurred through random selection, but some was due to convenience sampling when spouses, family, or friends asked to be in the same group due to transportation needs. The make-up of the groups could be seen as a limitation or as a benefit. The groups were not homogeneous as individuals had a variety of chronic health conditions. This could be seen as a benefit in that participants learned from each other and some even stated they did not feel as bad about their own situation when they saw what others were experiencing. It is also possible that not all individuals, or health conditions, would benefit at the same level. Finally, it is possible that response bias may have occurred as participants wanted to demonstrate positive responses. The researchers attempted to control for this; however, as they frequently reminded individuals that they wanted accurate responses, no matter what those responses might be.

## Conclusion

The broad problem addressed by this study was the need to improve the mood, health, resilience, and well-being of adults living with chronic health conditions and adapting current health programs to address these needs. In addition, a related problem addressed was improving individuals’ physical and mental health outcomes, well-being, mood, resilience, coping skills, stress, and health indicators while promoting behavior change. In addition, this study was designed to address the gap in the literature on the use of arts-based interventions to assist in improving population health, mood, resilience, and well-being. Our findings helped to demonstrate that arts-based programming, with an underlying theoretical framework of arts integration theory, can have a positive effect on the overall mood, health, resilience, and well-being of individuals with chronic health conditions. This supports previous research that was conducted utilizing the arts to improve such domains as depression, anxiety, socialization, identity, stress, mood, self-worth, coping, quality of life, resilience, mental health, physical health, pain, distress, and the various types of well-being. Although arts integration theory has been increasingly used outside of education, most of the literature is still within the educational field. Therefore, this study took the various tenets of arts integration theory, applied them to teaching topics such as improving mental and physical health, resilience, coping, mood, and various types of well-being. The arts were utilized for art’s sake, but also to increase learning of other concepts, change neural pathways, promote community, and increase brain processes.

Our results suggest that individuals who participated in the HeRe We Arts^®^ program developed new, or renewed, coping skills to assist them in dealing with their chronic health conditions. For some, this was dealing with pain or changes in physical abilities, and for others this was dealing with emotional distress or depression. These participants also benefited from the opportunity to socialize with each other, as well as the opportunity to have something to do that provided encouragement for them to leave their homes, thereby decreasing their isolation and depression. Based on these findings, arts-based programming should be utilized more frequently to maintain and improve public health; and further research should be conducted in order to analyze the outcomes of such programming.

## Data availability statement

The datasets presented in this article are not readily available because they require a DUA. Requests to access the datasets should be directed to gallagl@ccf.org.

## Ethics statement

The studies involving humans were approved by Cleveland Clinic Institutional Review Board. The studies were conducted in accordance with the local legislation and institutional requirements. The participants provided their written informed consent to participate in this study.

## Author contributions

LG, DB, and TS contributed to the conceptualization of the program and methodology, and assisted with leading the program and with data collection as needed. FB and MJ contributed to the methodology. LG reviewed the literature. IB performed formal statistical analysis. FB and LG reviewed and contributed to the data analysis. All authors contributed to the article and approved the submitted version.
